# Analysis of Individual High-Frequency Traders’ Buy–Sell Order Strategy Based on Multivariate Hawkes Process

**DOI:** 10.3390/e24020214

**Published:** 2022-01-29

**Authors:** Hiroki Watari, Hideki Takayasu, Misako Takayasu

**Affiliations:** 1 Department of Mathematical and Computing Science, School of Computing, Tokyo Institute of Technology, Yokohama 226-8502, Japan; watari.h.aa@m.titech.ac.jp; 2 Institute of Innovative Research, Tokyo Institute of Technology, Yokohama 226-8502, Japan; takayasu@csl.sony.co.jp; 3 Sony Computer Science Laboratories, Tokyo 141-0022, Japan

**Keywords:** high-frequency trader, multivariate Hawkes process, econophysics, forex market

## Abstract

Traders who instantly react to changes in the financial market and place orders in milliseconds are called high-frequency traders (HFTs). HFTs have recently become more prevalent and attracting attention in the study of market microstructures. In this study, we used data to track the order history of individual HFTs in the USD/JPY forex market to reveal how individual HFTs interact with the order book and what strategies they use to place their limit orders. Specifically, we introduced an 8-dimensional multivariate Hawkes process that included the excitations due to the occurrence of limit orders, cancel orders, and executions in the order book change, and performed maximum likelihood estimations of the limit order processes for 134 HFTs. As a result, we found that the limit order generation processes of 104 of the 134 HFTs were modeled by a multivariate Hawkes process. In this analysis of the EBS market, the HFTs whose strategies were modeled by the Hawkes process were categorized into three groups according to their excitation mechanisms: (1) those excited by executions; (2) those that were excited by the occurrences or cancellations of limit orders; and (3) those that were excited by their own orders.

## 1. Introduction

To gain a deeper understanding of the mechanisms of financial markets, it is necessary to clarify the order strategies of individual market participants. In financial markets, recent developments in information technology have made it possible to track the transactions of individual market participants in detail. These technological advances have led to the analysis of the trading strategies of individual market participants and how these strategies affect financial markets. For example, Odean [1], and Grinblatt and Keloharju [2], reported the relationship between historical returns and market participants’ decisions to buy and sell stocks. The position management strategies of individual market participants were analyzed based on the data, which confirmed that these strategies actually affected market prices in the near future [3]. Individual strategies for placing buy and sell orders in response to market price changes were approximated using a simple mathematical model, and the basic statistical properties of financial Brownian motion were theoretically derived based on the kinetic theory in a manner parallel to traditional statistical physics [4,5,6].

In particular, high-frequency traders (HFTs) have recently attracted attention. HFTs are algorithmic traders who can react to market changes in milliseconds and place or cancel, buy and sell orders at high frequencies [7]. Because of the development of information technology, they have a large presence in financial markets around the world. In fact, HFTs accounted for 68.3% of the total trading volume in the stock market [8]. Furthermore, HFTs currently account for the majority of orders shown in the order book [9,10,11]. The availability of high-frequency trading data has triggered the academic study of HFTs [12,13]. Previous studies have generally agreed that HFTs make market spreads smaller and enhance market liquidity [8,14,15]. As an indicator for predicting the short-term volatility of market prices from the order book information, the volume-synchronized probability of informed trading (VPIN) has been proposed and actively studied [16,17,18,19,20]. In addition, informed trading using the advantage of information such as public news and confidential information has been studied using high-frequency data [21,22,23,24,25]. We believe that it is crucial to gain a deep understanding of their trading behavior in current financial markets, where HFTs provide most of the liquidity.

In this study, we used a multivariate Hawkes process to investigate the processes used by individual HFTs for generating sell and buy limit orders in the USD/JPY forex market, and clarified when each HFT placed buy–sell limit orders. The Hawkes process is a type of non-homogeneous Poisson process proposed by Hawkes [26]. As will be explained later, it is characterized by an intensity function which determines the probability of the occurrence of an event in a point process. It utilizes an excitation term that is affected by past events, and can describe a point process associated with past events. Similar ideas have been independently introduced for financial markets to explain the strong correlation to past events, such as the “autoregressive conditional duration model” [27] and “self-modulation processes” [28]. The Hawkes process is a useful model for interpreting financial phenomena, in which many factors interact to produce complex aspects. In this paper, we show that it is also useful for interpreting the behavior of HFTs. Specifically, we introduced a multivariate Hawkes process in which the process of generating HFTs’ buy–sell limit orders is mutually excited by a total of eight events, such as the creation of limit orders, the cancellation of limit orders, and execution in the order book, showing that the order behaviors of many HFTs can be modeled by the Hawkes process.

Hawkes processes [29] have various applications in the financial field, such as those related to volatility clustering [30], market activity and risk [31,32,33], and market impact [34]. In particular, the Hawkes process has been actively employed as an approach to the dynamic description of order books, where a set of order types is specified and a multivariate Hawkes process is fitted to their timestamps [35,36,37,38,39,40,41]. However, there has been no study that used a multivariate Hawkes process to investigate the order generation processes of individual HFTs. In today’s financial markets, where the majority of order books are made up of HFTs’ orders, our empirical results provide new information from a more microscopic perspective. We believe that this study shed light on how HFTs provide liquidity to the market.

The remainder of this paper is organized as follows. Section 2 explains the datasets and describes the HFTs that were analyzed in this research. Section 3 introduces the multivariate Hawkes process and describes the method used for parameter estimation. In Section 4, a clustering analysis of 134 HFTs is introduced to categorize their strategies based on the estimated Hawkes’ parameters. In Section 5, we discuss our results.

## 2. Data

First, we provide a basic description of the order data for the USD/JPY forex market (EBS market), along with individual trader IDs (see Section 2.1). We then define the HFTs in this market (see Section 2.2) and show some examples to explain how their buy–sell limit order generation is linked to changes in the order book (see Section 2.3).

### 2.1. EBS Market Data Description

In this study, we used high-frequency data for the USD/JPY forex market provided by the EBS. EBS is an interbank forex market and one of the largest financial platforms in the world. Because it is an interbank market, most market participants are professional traders from banks, hedge funds, and other financial institutions, and our forex dataset contains their trading data. Our dataset contains information from five days (from 21:00 GMT on 5 June 2016 to 21:00 GMT on 10 June 2016), with a total of approximately 2.8 million orders and a transaction volume of USD 68 billion corresponding to this period. Table 1 shows an example of the raw data we used. The data for each of the 2.8 million orders contained not only the order type, price, volume, and timestamp (in milliseconds), but also an anonymized trader ID that could identify who submitted the order. Using these trader IDs, we could track individual traders’ full orders in milliseconds. In addition, the minimum price unit that a trader could submit was JPY 0.005, and the minimum transmission volume was USD 1 million.

**Figure 1:**
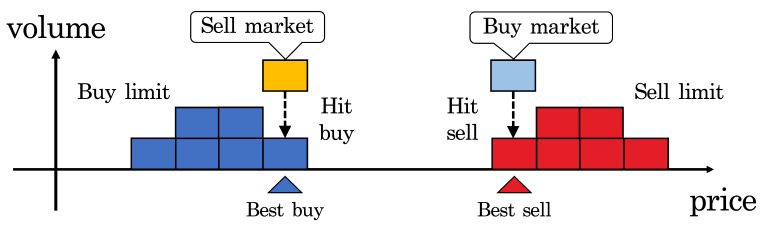
Schematic of trading in order book. In the EBS market, even a sell (buy) limit order becomes a hit buy (sell) if a buy (sell) limit order at the same price is already in the order book.

The EBS market is open 24 h a day from Monday morning to Friday at midnight, and trading is conducted via a double auction system in the order book. Figure 1 shows a schematic of the trading in the order book, where the horizontal axis is the price and the vertical axis is the volume. There are six order types for trading: buy/sell limit orders, buy/sell cancel orders, and buy/sell market orders. Limit orders are submitted at the trader’s desired price and remain in the order book until traded or cancelled. Cancel orders are submitted by a trader to cancel a limit order that they previously submitted. Market orders are submitted at the current best limit price. Transactions that are executed by buy market orders are called hit sell transactions, and transactions executed by sell market orders are called hit buy transactions (see Figure 1). If the best price worsens (e.g., the best sell limit price becomes higher) before the market receives the market order at the best price, the market order is automatically invalidated. In fact, this study found that 79.5% of the market orders were invalidated without being executed.

**Figure 2:**
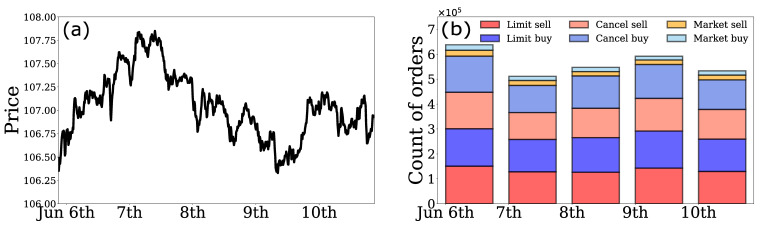
(**a**) Average trading price per 10 min window; (**b**) daily number of orders for the 6 types of orders in unit of $10^{5}$.

Figure 2a shows the average trading price per 10 min window over the 5 days we analyzed. During this period, there are no market crashes or spikes. Figure 2b shows the number of each type of order per day, which looks stable.

### 2.2. Definition of HFTs

“HFTs” is a general term for traders who place and cancel orders at high speed and high frequency according to an algorithm, but there are various definitions. In this study, we define an HFT as a trader who places both buy and sell limit orders and presents an average of 500 or more limit orders per day following the previous report written by a researcher from EBS [9]. Based on this definition, the number of HFTs was 134 out of the 1031 traders included in this 5-day data set. These 134 HFTs accounted for 89.6% of the market’s total number of limit orders.

**Figure 3:**
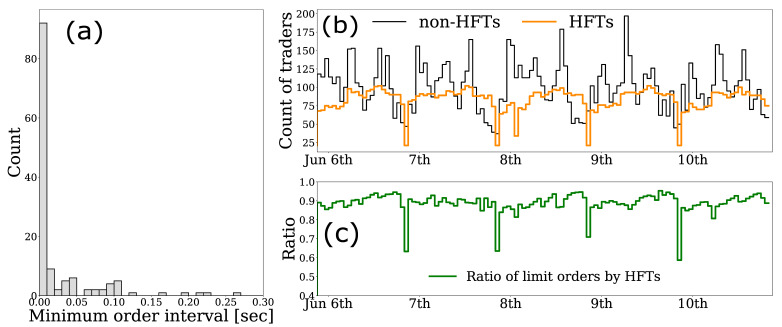
(**a**) Histogram of the minimum time intervals between orders for 134 HFTs individually; (**b**) hourly changes in the number of HFTs and non-HFTs participating in trading; and (**c**) hourly change of the percentage of limit orders provided by HFTs.

Figure 3a shows the histogram of the minimum time interval between orders for each HFT. There is no description in the data to identify whether the ID is a human or a computer; however, Figure 3a shows that most of the intervals are within 0.1 s, which are difficult for a human to execute.

In Figure 3b, we plot the number of HFTs and non-HFTs participating in the market every hour, indicating that the number of HFTs is relatively stable compared to non-HFTs. Figure 3c shows the percentage of limit orders placed by HFTs every hour, demonstrating that the majority of the limit orders are provided by the HFTs.

### 2.3. Basic Properties of HFTs

**Figure 4:**
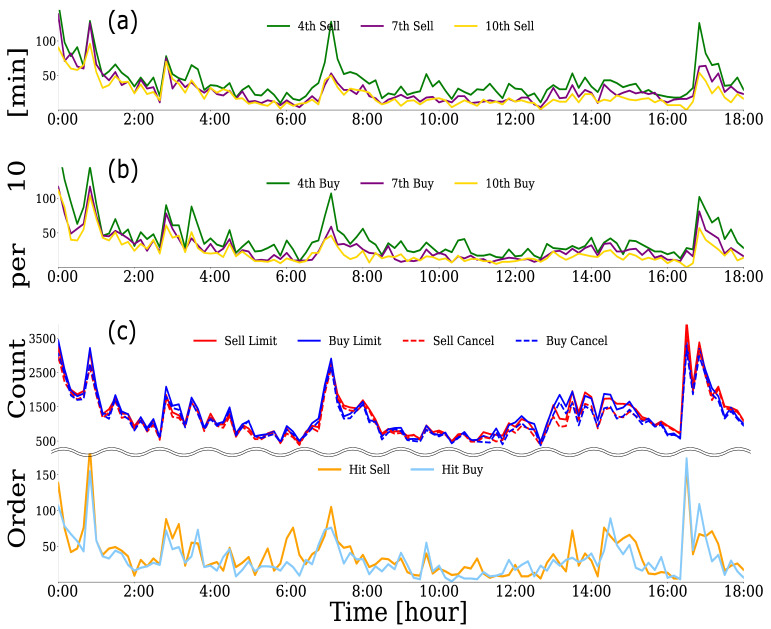
Numbers of (**a**) sell limit orders and (**b**) buy limit orders per 10 min window for three HFTs (green: HFT with 4th highest order frequency; purple: HFT with 7th highest order frequency; yellow: HFT with 10th highest order frequency). (**c**) Numbers for six types of orders per 10 min window in the order book (red: sell limit order; blue: buy limit order; red dotted line: sell cancel; blue dotted line: buy cancel; orange: hit sell; sky blue: hit buy). The vertical axis of each figure shows the number of each type of order per 10 min window, and the horizontal axis shows the time from 0:00 to 18:00 on 6 June 2016.

In this study, we focused on the limit order generation process of HFTs, which accounted for the majority of limit orders in the order book. Naturally, the order strategies (i.e., the processes used to submit limit orders) of HFTs differed from every algorithm. However, it is natural for them to see the quotes in the order book when submitting their limit orders. Figure 4a,b plot the numbers of buy–sell limit orders per 10 min window for three HFTs, respectively, and Figure 4c plots the numbers for six types of orders in the order book. From Figure 4, we can observe that the numbers of buy–sell limit orders from the three HFTs increased or decreased simultaneously and tended to be in sync. More interestingly, the numbers of these HFTs’ buy–sell limit orders tend to be in sync with the numbers for each type of order in the order book where all market participants’ orders are submitted. Since the above synchronization phenomenon was confirmed for many HFTs, we believe that many HFTs react instantaneously to some changes in the market when submitting limit orders.

## 3. Method

The preceding section showed that the limit order generation processes of the HFTs tended to be in sync with traders’ orders or orders in the order book. To clarify how 134 HFTs’ buy–sell limit orders react to the other order events, their order processes are modeled by the multivariate Hawkes process. In this section, we introduce the multivariate Hawkes process that we used in this study (see Section 3.1) and explain the parameter estimation method based on maximum likelihood estimation (see Section 3.2). We then describe the validity of the estimation results (see Section 3.3).

### 3.1. Model

This section presents an overview of the Hawkes process and introduces our model.

#### 3.1.1. Mathematical Notation

Let us consider point process $\left\{t_{i}\right\}$, which is a sequence of non-negative random variables such that ∀i∈N, $t_{i}<t_{i+1}$. For point process $\left\{t_{i}\right\}$, the conditional intensity function is defined as follows [42]:

(1) $$\lambda \left(t|H_{t}\right)=\lim_{\Delta t\to 0}\frac{P\left\{N\left(t+\Delta t\right)-N\left(t\right)=1|H_{t}\right\}}{\Delta t}$$

where P{A|B} represents the probability of *A* under condition *B*, N(t) is the cumulative number of event occurrences at time *t* (i.e., a counting process), and $H_{t}$ is the history of the process up to time *t* containing a sequence of event times $\left\{t_{i}\right\}$ (i.e., a filtration). As can be seen from the definition, $\lambda \left(t|H_{t}\right)\Delta t$ represents the probability of an event occurring in time interval [t,t+Δt). Here, we use the shorthand notation $\lambda \left(t\right)\equiv \lambda \left(t|H_{t}\right)$, assuming the history up to time *t*, and we call the Poisson parameter λ(t) an intensity function.

#### 3.1.2. Overview of Hawkes Process

The Hawkes process is a point process in which the intensity function is affected by the occurrence of past events. Let $\left\{t_{i}\right\}$ be a point process, and N(t) be the associated counting process, and the intensity function of the generalized Hawkes process is defined as follows [43]:

(2) $$\lambda \left(t\right)=c+\int_{-\infty }^{t}\phi \left(t-s\right)dN\left(s\right)=c+\sum_{t_{i}<t}\phi \left(t-t_{i}\right)$$

where *c* is a positive constant showing a base intensity, and ϕ(t) is a kernel function that expresses the effect of event $t_{i}$ from the past on the current intensity [44]. There are various types of kernel functions, and their properties have been well studied. In this study, we applied an exponential kernel $\alpha e^{-\beta t}$, which is a popular kernel function that was originally proposed by Hawkes [26]. We call this Hawkes process a univariate Hawkes process because it is affected by its own events.

The Hawkes process can be extended to a multivariate model in which several types of point processes interact with each other. Let $\left\{t_{i}\right\}\equiv \left\{\left\{t_{1,i}\right\},\left\{t_{2,i}\right\},\ldots ,\left\{t_{M,i}\right\}\right\}$ be M-variable point processes, and $N\left(t\right)=\{N_{1}\left(t\right),N_{2}\left(t\right),\ldots ,N_{M}\left(t\right)\}$ be the associated counting process, the intensity function of a multivariate Hawkes process for point process $\left\{t_{n,i}\right\}$ is defined as follows [43]:

(3) $$\lambda_{n}\left(t\right)=c_{n}+\sum_{m=1}^{M}\int_{-\infty }^{t}\phi_{n,m}\left(t-s\right)dN_{m}\left(s\right)=c_{n}+\sum_{m=1}^{M}\sum_{t_{m,i}<t}\phi_{n,m}\left(t-t_{m,i}\right)$$

As in the case of the univariate Hawkes process, $c_{n}$ is a positive constant showing a base intensity, and $\phi_{n,m}\left(t\right)$ is a kernel function that expresses the effect of event $t_{m,i}$ from the past on the current intensity. In this case, $\phi_{n,m}\left(t\right)=\alpha_{n,m}e^{-\beta_{n,m}t}$, with positive constant parameters $\alpha_{n,m}$ and $\beta_{n,m}$, and the intensity function for point process $\left\{t_{n,i}\right\}$ is given as follows:

(4) $$\begin{array}{ccc} \lambda_{n}\left(t\right) & = & c_{n}+\sum_{m=1}^{M}\int_{-\infty }^{t}\alpha_{n,m}e^{-\beta_{n,m}\left(t-s\right)}dN_{m}\left(s\right)\\ & = & c_{n}+\sum_{m=1}^{M}\sum_{t_{m,i}<t}\alpha_{n,m}\exp(-\beta_{n,m}\left(t-t_{m,i}\right)) \end{array}$$

**Figure 5:**
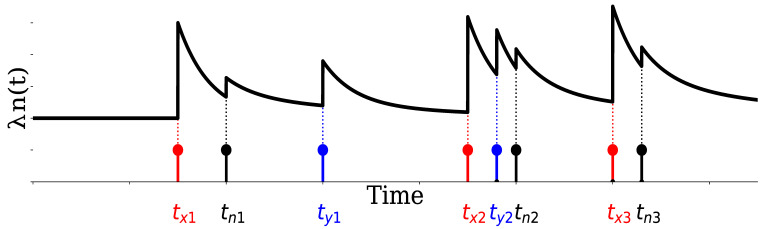
Schematic showing an example of the time evolution of the intensity function for a multivariate Hawkes process with the exponential kernel $\phi_{n,m}\left(t\right)=\alpha_{n,m}e^{-\beta_{n,m}t}$.

Figure 5 is a schematic of this intensity function (Equation (4)). The intensity function, $\lambda_{n}\left(t\right)$, increases $\alpha_{nm}$ at event time $t_{n,i}$ and exponentially decays with a time constant of $1/\beta_{nm}$. Thus, $\lambda_{n}\left(t\right)$ is excited not only by its own events, but also by other events, and the multivariate Hawkes process can represent such mutual interactions.

The quantity, $\rho_{n,m}$, expressed in the following equation (Equation (5)) in the case of an exponential kernel is called the branching ratio [45,46]. This is the expectation of the number of occurrences of event *n* caused by the occurrence of event *m*. A larger value for this number represents a greater impact of event *m* on event *n*, and this value is an important quantity for interpreting the Hawkes process:

(5) $$\rho_{n,m}\equiv \frac{\alpha_{n,m}}{\beta_{n,m}}=\int_{t_{m,i}}^{\infty }\alpha_{n,m}\exp\left(-\beta_{n,m}\left(t-t_{m,i}\right)\right)dt$$

#### 3.1.3. Trader Model

In this study, the buy and sell limit order processes of 134 HFTs are modeled by eight-variable Hawkes processes with exponential kernels that are excited by a total of eight-point processes. These eight types were the target HFTs’ sell limit (TS) and buy limit (TB), and six types of orders in the order book: sell limit (SL); buy limit (BL); sell cancel (SC); buy cancel (BC); hit sell (HS); and hit buy (HB).

Let $\left\{t_{i}\right\}\equiv \left\{\left\{t_{TS,i}\right\},\left\{t_{TB,i}\right\},\left\{t_{SL,i}\right\},\left\{t_{BL,i}\right\},\left\{t_{SC,i}\right\},\left\{t_{BC,i}\right\},\left\{t_{HS,i}\right\},\left\{t_{HB,i}\right\}\right\}$ be an eight-variable point process, the intensity functions for $\left\{t_{TS,i}\right\}$ and $\left\{t_{TB,i}\right\}$ are given as follows:

(6) $$\lambda_{TS}\left(t\right)=c_{TS}+\sum_{m\in M}\sum_{t_{m,i}<t}\alpha_{TS,m}\exp(-\beta_{TS,m}\left(t-t_{m,i}\right))$$

(7) $$\lambda_{TB}\left(t\right)=c_{TB}+\sum_{m\in M}\sum_{t_{m,i}<t}\alpha_{TB,m}\exp(-\beta_{TB,m}\left(t-t_{m,i}\right))$$

where M≡{TS,TB,SL,BL,SC,BC,HS,HB}. We assume that the above intensity functions (Equations (6) and (7)) represent the buy and sell limit order processes of each HFT and examine which events affect their order generation processes. Hereafter, the abbreviations listed in Table 2 are used for the order events.

### 3.2. Parameter Estimation Using Maximum Likelihood Estimation

For Equations (6) and (7), we apply the maximum likelihood estimation method for the parameter estimation. Because the functional forms of the intensity functions are the same for $\left\{t_{TS,i}\right\}$ and $\left\{t_{TB,i}\right\}$, we solve the log-likelihood functions in the following manner. For the point process $\left\{t_{TS,i}\right\}$, the log-likelihood function in time interval [0,T] is given by the following [47]:

(8) $$\begin{array}{c} \log L\left(c_{TS},\alpha_{TS},\beta_{TS}\right)=-\int_{0}^{T}\lambda_{TS}\left(t\right)dt+\sum_{i=1}^{n}\log\lambda_{TS}\left(t_{i}\right)\\ =-c_{TS}T+\sum_{m\in M}\frac{\alpha_{TS,m}}{\beta_{TS,m}}\left[\sum_{t_{m,i}<T}\{exp\left(-\beta_{TS,m}\left(T-t_{m,i}\right)-1\right\}\right]\\ +\sum_{i=1}^{n}\log\left[c_{TS}+\sum_{m\in M}\sum_{t_{m,j}<t_{TS,i}}\alpha_{TS,m}exp\left(-\beta_{TS,m}\left(t_{TS,i}-t_{m,j}\right)\right)\right] \end{array}$$

The same formulation is also applied for $\log L(c_{TB},\alpha_{TB},\beta_{TB})$.

These log-likelihood functions are differentiable by each parameter, and we optimized them by Adam [48], which is a type of gradient descent method, to obtain the maximum likelihood estimators. Here, the initial values are $(c_{TS},\alpha_{TS},\beta_{TS})=$$\left(c_{TB},\alpha_{TB},\beta_{TB}\right)$ = (0.1,0.1,10), and the various parameters required for Adam are set following the values in the original reference [48]. Here, $\alpha_{TS}$, $\beta_{TS}$, $\alpha_{TB}$, and $\beta_{TB}$ are vectors of eight variables (e.g., $\alpha_{TS}=\left(\alpha_{TS,TS},\alpha_{TS,TB},\ldots ,\alpha_{TS,HS}\right)$).

It is also known that the computation of the gradients of the log-likelihood function of the Hawkes process usually requires the computation of $O(N^{2})$. However, using a recursive formulation that can be used when the kernel function is an exponential function, we perform maximum likelihood estimation with O(N) computational complexity (see Ogata [49] for the recursive formulation).

In addition, the HFTs do not always continuously place orders. Therefore, if an HFT did not place any orders for more than 15 min, we considered such period as not participating in the trade and performed the maximum likelihood estimation for each trader by ignoring such inactive periods.

### 3.3. Validity of Estimation Results

Based on a residual analysis [50], we assessed the goodness-of-fit of the point process model. Let the intensity function for point process $\left\{t_{i}\right\}$ be λ(t), then the sequence, $\left\{\tau_{i}\right\}$, of random variables, where each element is transformed by $\tau_{i}\equiv \int_{0}^{t_{i}}\lambda \left(t\right)dt$, has the distribution of a stationary Poisson process with intensity 1, and the transformed residual $\Delta \tau_{i}\equiv \tau_{i+1}-\tau_{i}$ has an exponential distribution with the unit mean.

Therefore, if the estimated HFT intensities, ${\hat{\lambda }}_{TS}\left(t\right)$ and ${\hat{\lambda }}_{TB}\left(t\right)$, are good approximations of the true intensities, $\lambda_{TS}\left(t\right)$ and $\lambda_{TB}\left(t\right)$, respectively, then the transformed residuals, $\Delta {\hat{\tau }}_{TS,i}$ and $\Delta {\hat{\tau }}_{TB,i}$, are expected to follow the exponential distributions with the unit mean. Here, the transformed residuals are defined as $\Delta {\hat{\tau }}_{TS,i}\equiv \int_{t_{TS,i}}^{t_{TS,i+1}}{\hat{\lambda }}_{TS}\left(t\right)dt$ and $\Delta {\hat{\tau }}_{TB,i}\equiv \int_{t_{TB,i}}^{t_{TB,i+1}}{\hat{\lambda }}_{TB}\left(t\right)dt$, respectively, which can be derived in the case of $\Delta {\hat{\tau }}_{TS,i}$ as an example (Equation (9)) because the intensity function has the same form as described above:

(9) $$\begin{array}{c} \Delta {\hat{\tau }}_{TS,i}={\hat{c}}_{TS}\Delta t_{TS,i}-\sum_{m\in M}\sum_{t_{m,i}<t_{TS,i+1}}\frac{{\hat{\alpha }}_{n,m}}{{\hat{\beta }}_{n,m}}(exp\left(-{\hat{\beta }}_{TS,m}\left(t_{TS,i+1}-t_{m,i}\right)\right)\\ -exp(-{\hat{\beta }}_{TS,m}\left(t_{TS,i}-t_{m,i}\right))) \end{array}$$

**Figure 6:**
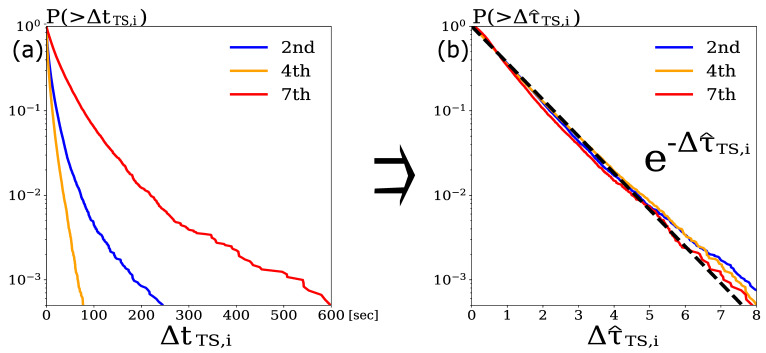
Cumulative distributions of $\Delta t_{TS,i}$ (**a**) and $\Delta {\hat{\tau }}_{TS,i}$ (**b**) for three typical HFTs (blue: HFT with 2nd highest order frequency; orange: HFT with 4th highest order frequency; and red: HFT with 7th highest order frequency).

Figure 6a shows the cumulative distribution of the three HFTs’ original residuals, $\Delta t_{TS,i}$, and Figure 6b shows the cumulative distribution of the same three HFTs’ transformed residuals, $\Delta {\hat{\tau }}_{TS,i}$. We confirmed that the transformed residuals for the three HFTs approximately followed the exponential distribution with the unit mean, which implied that the intensities of $\left\{t_{TS,i}\right\}$ were properly approximated. Because not all of the HFTs’ order generation processes could be modeled by the Hawkes process proposed here, the above operations were performed for the sell and buy order processes of the 134 HFTs.

Here, we apply the Kullback–Leibler divergence between the distribution of the transformed residuals and the exponential distribution with the unit mean as a criterion to determine whether the approximations of the intensities of each HFTs’ $\left\{t_{TS,i}\right\}$ and $\left\{t_{TB,i}\right\}$ are appropriate. Since we estimate the intensity functions of the two-point processes for each trader, we calculate the sum of the respective Kullback–Leibler divergences ($D_{TS+TB}^{KL}$), as defined by Equation (10) below:

(10) $$D_{TS+TB}^{KL}=\sum_{j}p_{j}\left(\Delta {\hat{\tau }}_{TS,i}\right)\log\frac{p_{j}\left(\Delta {\hat{\tau }}_{TS,i}\right)}{q_{j}\left(\Delta {\hat{\tau }}_{TS,i}\right)}+\sum_{j}p_{j}\left(\Delta {\hat{\tau }}_{TB,i}\right)\log\frac{p_{j}\left(\Delta {\hat{\tau }}_{TB,i}\right)}{q_{j}\left(\Delta {\hat{\tau }}_{TB,i}\right)}$$

Here, $q_{j}\left(\tau \right)$ is the discrete exponential distribution with the unit mean, and $p_{j}\left(\tau \right)$ is the sampling distribution. The bin size of the discrete distribution is assumed to be 1. The threshold of accepting $D_{TS+TB}^{KL}$ error will be determined in the next section.

## 4. Results

In this section, we first show that the Hawkes process introduced here successfully approximated the intensity of the limit order generation process for 104 of the 134 HFTs by applying the method described in Section 3.3 (see Section 4.1). We then categorize the order generation processes of the 104 successfully estimated HFTs into three groups according to their excitation mechanisms, and explain how each group of HFTs places their orders (see Section 4.2).

### 4.1. $D_{TS+TB}^{KL}$ Calculation Results for All HFTs

**Figure 7:**
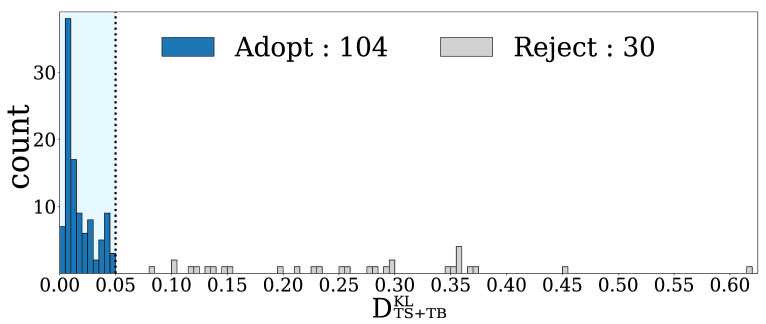
A histogram of $D_{TS+TB}^{KL}$ values for all 134 HFTs. Out of 134 HFTs, 104 fell within the acceptable error threshold of 0.05, and the remaining 30 HFTs exceeded the threshold.

Figure 7 shows a histogram of the $D_{TS+TB}^{KL}$ values for the 134 HFTs. From the histogram, there are many HFTs whose values of $D_{TS+TB}^{KL}$ are very close to 0 and the plots are scattered for $D_{TS+TB}^{KL}>0.05$, so we set the threshold as 0.05. The 104 HFTs who fell into the range of $D_{TS+TB}^{KL}<0.05$ were considered to be traders whose order generation processes were well modeled, while the rest of the 30 HFTs who fell into the range of $D_{TS+TB}^{KL}\geq 0.05$ were considered to be traders whose order generation processes were poorly modeled by the Hawkes process introduced here.

**Figure 8:**
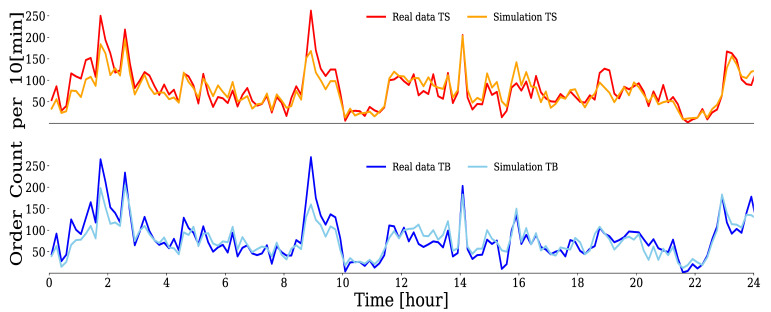
Comparison between simulations and real data of $\left\{t_{TS,i}\right\}$ and $\left\{t_{TB,i}\right\}$ for HFT with $D_{TS+TB}^{KL}=0.0238$. The horizontal axis represents the time over a 24 h period, and the vertical axis represents the number of order occurrences per 10 min window.

With the estimated parameters, the Hawkes process could be simulated using the thinning method [51]. For example, Figure 8 compares the time series of the number of orders per 10 min window for the real data of an HFT with $D_{TS+TB}^{KL}=0.0238$ and a simulated time series. It can be confirmed that both sell limit orders (upper figure) and buy limit orders (lower figure) successfully reproduce the behavior of the real data.

**Figure 9:**
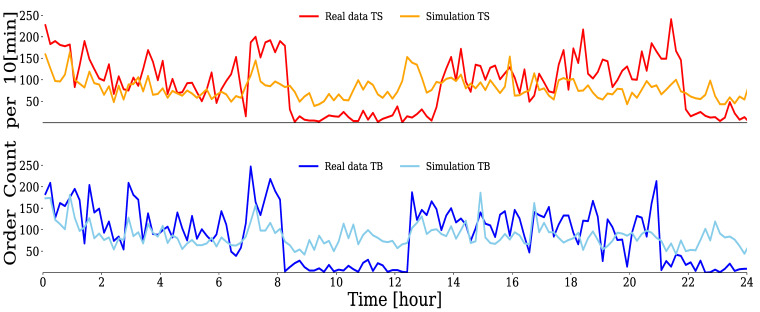
Comparison between simulations and real data of $\left\{t_{TS,i}\right\}$ and $\left\{t_{TB,i}\right\}$ for HFT with $D_{TS+TB}^{KL}=0.211$. The horizontal axis represents the time during a 24 h period, and the vertical axis represents the number of order occurrences per 10 min window.

On the other hand, Figure 9 shows a comparison of the simulated and real data for an HFT with $D_{TS+TB}^{KL}=0.211$, which was judged not to be properly estimated by the Hawkes process. It can be seen that the deviations from the real data for both sell limit orders (upper figure) and buy limit orders (lower figure) are larger than in the case of Figure 8. The Hawkes process introduced here did not adequately explain the order generation process for this trader with a large error, $D_{TS+TB}^{KL}$. Because some traders could not be modeled by the Hawkes process, in the following, we report the results of our clustering analysis of the order generation processes of 104 HFTs after excluding 30 traders.

### 4.2. Results of Clustering Analysis

Here, we categorize the 104 HFTs whose limit order generation process was properly estimated according to the similarity of their excitation mechanisms (the branching ratio), and describe how each group of HFTs placed buy–sell limit orders and provided liquidity to the market. As defined in Equation (5) in Section 3.3, the branching ratio, $\rho_{n,m}$, is an absolute value that represents the expectation for the number of occurrences of event *n* caused by the occurrence of event *m*. To evaluate the excitations of $\left\{t_{TS,i}\right\}$ and $\left\{t_{TB,i}\right\}$ relative to each HFT, we introduced normalized branching ratios ${\bar{\rho }}_{TS,m}$ and ${\bar{\rho }}_{TB,m}$, which are defined by the following equations, so that the sum is equal to 1:

(11a) $$\begin{array}{cc} {\bar{\rho }}_{TS,m} & \equiv \frac{\rho_{TS,m}}{\sum_{i\in M}\rho_{TS,i}} \end{array}$$

$$\begin{array}{cc} {\bar{\rho }}_{TB,m} & \equiv \frac{\rho_{TB,m}}{\sum_{i\in M}\rho_{TB,i}} \end{array}$$

**Figure 10:**
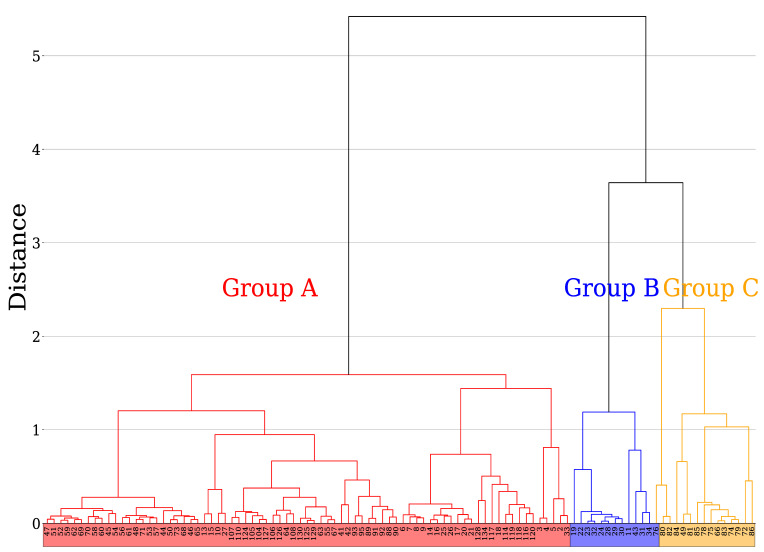
Dendrogram based on the Ward method of clustering for 104 HFTs that were successfully modeled by the Hawkes process. The vertical axis represents the distance between the clusters, as defined in Equation (12), and the horizontal axis shows the labels of the HFTs according to the order frequency (red: Group A with 77 HFTs; blue: Group B with 12 HFTs; yellow: Group C with 15 HFTs).

Because both $\left\{t_{TS,i}\right\}$ and $\left\{t_{TB,i}\right\}$ are 8-variable Hawkes processes, 16 normalized branching rates were defined for each HFT. Figure 10 shows a dendrogram of the hierarchical clustering of the 104 HFTs using these 16 variables. For this hierarchical clustering, we used the Ward method [52] to join clusters in the order of the decreasing sum of squares after joining. The vertical axis in Figure 10 represents the distance between clusters with an increase in the sum of squares when clusters *A* and *B* are joined, and is defined by the following equation:

$$\Delta \left(A,B\right)=\sum_{i\in A\cup B}{∥{\vec{x}}_{i}-{\vec{m}}_{A\cup B}∥}^{2}-\sum_{i\in A}{∥{\vec{x}}_{i}-{\vec{m}}_{A}∥}^{2}-\sum_{i\in B}{∥{\vec{x}}_{i}-{\vec{m}}_{B}∥}^{2}\left(12\right)$$

where ||d|| denotes the Euclidean distance, and ${\vec{m}}_{j}$ is the center of cluster *j*.

Based on the distance between the clusters, we found it reasonable to categorize the HFTs into three groups with the threshold distance around 3, as shown in Figure 10, and designated them as Group A, Group B, and Group C. There were 77 HFTs in Group A, 12 in Group B, and 15 in Group C. The number of clusters becomes larger for a lower threshold distance, however, we confirmed that properties of any smaller groups are quite similar to one of these three groups in the graphical representation of an interaction network to be discussed in the following.

The remainder of this section explains the order events that excited the HFTs in each group to place buy–sell limit orders based on the estimated Hawkes parameters.

#### 4.2.1. Group A

**Figure 11:**
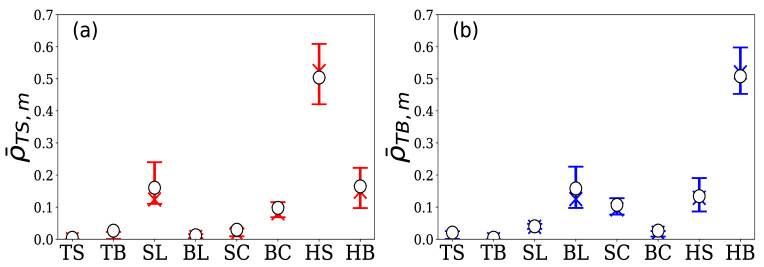
Percentile plot of normalized branching ratios (**a**) ${\bar{\rho }}_{TS,m}$ and (**b**) ${\bar{\rho }}_{TB,m}$ for 77 HFTs in group A. The vertical axis represents the normalized branching ratios by event *m*, and the horizontal axis represents element m∈M in both figures (top bar: 75th percentile; X symbol: median; bottom bar: 25th percentile; ◯ symbol: the mean).

Group A is comprised of 77 HFTs. The total number of limit orders in the 5 days was approximately 850,000, which accounted for 62.5% of the total number of limit orders in the market. Figure 11 shows the quartiles and means of the 16 normalized branching ratios for these 77 HFTs, where (a) represents ${\bar{\rho }}_{TS,m}$ and (b) represents ${\bar{\rho }}_{TB,m}$. From Figure 11a, it can be seen that the generation of sell limit orders by the HFTs in Group A was most excited by hit sell, which greatly exceeded the excitation from other events. In contrast, Figure 11b shows that their buy limit order generation was most excited by hit buy, which also greatly exceeded the excitation from other events.

**Figure 12:**
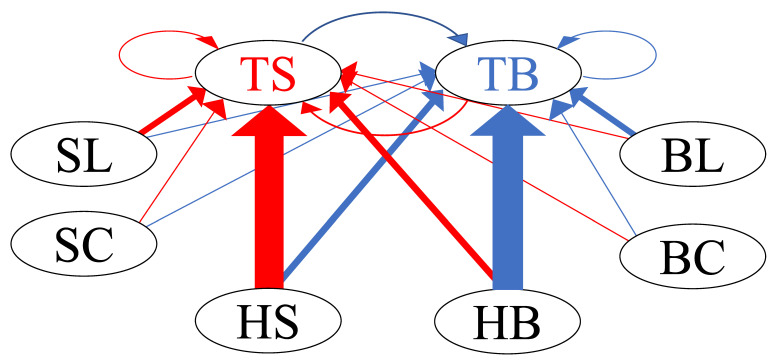
Network graph of interaction between buy–sell limit orders of HFTs in Group A and all types of orders in the order book.

Figure 12 illustrates the network graph of the buy and sell limit orders of the HFTs in Group A, along with all types of orders, using these normalized branching ratios. The size of the directed edges of the network is proportional to the mean value of the normalized branching ratio, e.g., edges directed from HS to TS and from HB to TB represent strong excitations.

In addition, because the kernel function of the Hawkes process is an exponential function, the time constant, which is a measure of the response speed of one excitation at a time, is given by $\beta_{n,m}^{-1}$. The mean values of the estimated time constant for the HFTs in Group A are summarized in Table 3. It is suggested that their reaction speed to an event is approximately 0.1 s, which is reasonable for HFTs who trade at very high speeds.

#### 4.2.2. Group B

**Figure 13:**
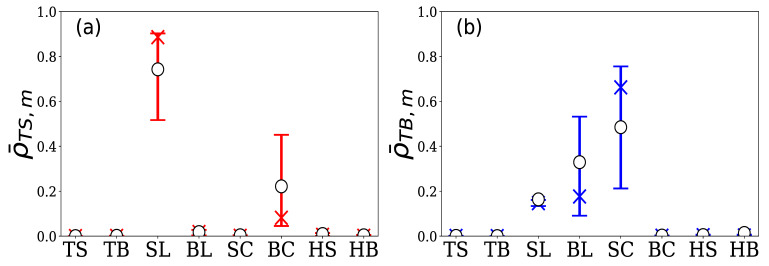
Percentile plot of normalized branching ratios (**a**) ${\bar{\rho }}_{TS,m}$ and (**b**) ${\bar{\rho }}_{TB,m}$ for 12 HFTs in Group B. The vertical and horizontal axes are the same as those in Figure 10 (top bar: 75th percentile; X symbol: median; bottom bar: 25th percentile; ◯ symbol: the mean).

Group B is comprised of 12 HFTs. The total number of limit orders in the 5 days was approximately 174,000, which accounted for 12.7% of the total number of limit orders in the market. Figure 13 shows the quartiles and means of the 16 normalized branching ratios for these 12 HFTs. From Figure 13a, we can see that their sell limit order generation was excited by sell limit and cancel buy, and from Figure 13b, we can see that their buy limit order generation was excited by buy limit and cancel sell. Unlike Group A, they did not react to execution events but were excited by the generation and cancellation of limit orders in the order book.

**Figure 14:**
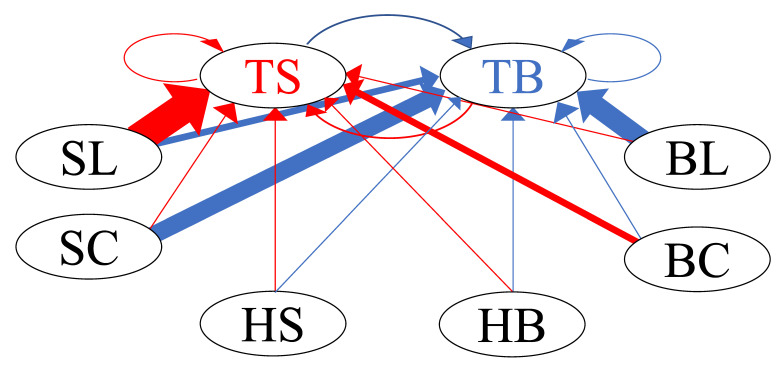
Network graph of interaction between the buy–sell limit orders of HFTs in Group B and all types of orders in the order book.

Figure 14 shows the interaction network of the buy and sell limit orders of the HFTs in Group B, along with all types of orders, using the mean of the normalized branching ratios, as in Figure 12.

The mean values of the time constants for each event are summarized in Table 4. As in the case of HFTs in Group A, these values suggest that the reaction speed to events were to be measured in milliseconds.

#### 4.2.3. Group C

**Figure 15:**
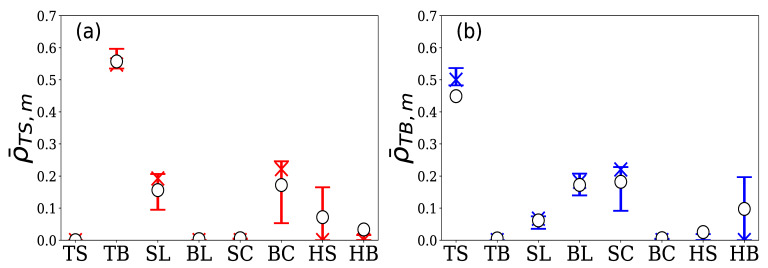
Percentile plot of normalized branching ratios (**a**) ${\bar{\rho }}_{TS,m}$ and (**b**) ${\bar{\rho }}_{TB,m}$ for 15 HFTs in Group C. The vertical and horizontal axes are the same as those in Figure 10 and Figure 11 (top bar: 75th percentile; X symbol: median; bottom bar: 25th percentile; ◯ symbol: the mean).

Group C is comprised of 15 HFTs. Their total number of limit orders in the 5 days was approximately 95,000, which accounted for 6.9% of the total number of limit orders in the market. From Figure 15a, it can be seen that the sell limit order generation of the HFTs in Group C was most strongly excited by their own buy limit, and was also excited by the sell limit and cancel buy. On the other hand, Figure 15b shows that their buy limit order generation was most strongly excited by their own sell limit, but was also excited by buy limit and cancel buy.

**Figure 16:**
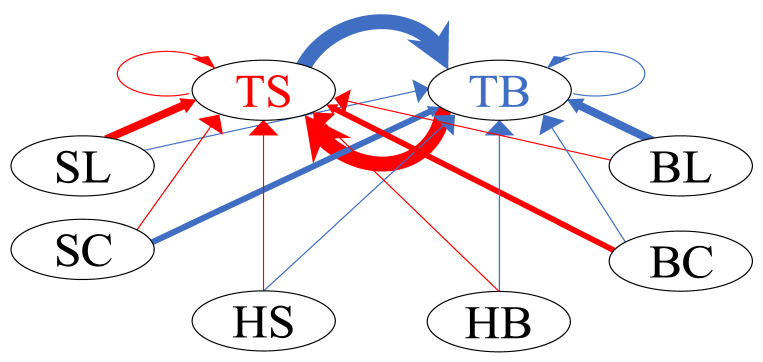
Network graph of interaction between buy–sell limit orders of HFTs in Group C and all types of orders in the order book.

Figure 16 shows the interaction network of the buy–sell limit orders of the HFTs in Group C, along with all types of orders, as in Figure 12 and Figure 14. The HFTs’ sell/buy limit orders interacted with each other.

The mean values of the time constants for each event are summarized in Table 5. The time constants of the excitations from TS to TB and from TB to TS were larger than 10 s, suggesting that the excitations were sustained for a very long time compared to those previously observed.

## 5. Conclusions

In summary, we introduced a multivariate Hawkes process to model the limit order generation processes of individual HFTs participating in the USD/JPY foreign exchange market for 5 days and analyzed their limit order generation mechanisms. First, we confirmed that an eight-variable Hawkes process, which consisted of each HFTs’ own buy–sell limit orders and the six types of orders in the order book, could adequately model the limit order generation processes of 104 of the 134 HFTs. Then, we categorized the 104 properly modeled HFTs into three categories based on the similarity of the excitation mechanisms measured by the parameter values of the Hawkes process. As a result, we confirmed that the majority of the HFTs in our dataset reacted to the execution of trades, while 12 of the 134 HFTs only reacted to limit orders and 15 of the 134 HFTs reacted to their orders. By evaluating the time constants of the estimated excitations of individual HFTs, we found that many HFTs responded to the most recent change in the order book in a very short time, by placing or canceling new orders. Since HFTs currently account for the majority of limit orders shown in the order book, the results of this analysis provide more microscopic insight into the dynamics of the order book than previous studies.

The following issues will be studied in the future as a generalization of the present work. The first goal is to clarify the limit order generation processes of the remaining 30 HFTs who could not be adequately modeled by the present analysis. The Hawkes process adopted in this study only included the impact of the occurrence of a recent order event and ignored important financial market influences such as the volume of orders, market price fluctuations and trends, and the positions of the HFTs. We believe that the information ignored in this study could contain variables that would explain their order generation processes. Second, although this study only focused on the generation of limit orders by HFTs, it is also important to clarify the cancellation process for limit orders by HFTs and the generation of market orders. Third, we did not pay attention to profit and loss; however, practically, a key factor in an HFT strategy is the ability to make stable profits.

As the period of our data is very short, we did not observe any abnormal behavior in the market; however, we cannot deny the possibility that HFTs may overreact and result in serious synchronization during other periods or in other markets. Further studies of the relations among Hawkes parameters and the case of crashes are needed to prevent the excessive synchronization of biased orders of buy or sell. Our results are important since the model we derived in this paper provides a foundation for performing such studies through simulations. HFTs play a central role in providing liquidity to the market, and further detailed analyses of HFT strategies will contribute to the development of modern financial markets in general.

## Figures and Tables

**Table 1:** Examples of raw data. Each order datum is tied to an anonymized trader ID.

Date	Order Time	Trader ID	Order Type	USD/JPY	Volume	Deal Time
5 June 2016	21:00:12.946	578	Sell limit	106.515	1	–
5 June 2016	21:01:13.647	HT6	Buy cancel	105.390	2	–
5 June 2016	21:02:20.148	JR1	Buy limit	105.405	1	21:02:20.499
5 June 2016	21:02:20.499	HSH	Sell market	105.405	1	21:02:20.499
5 June 2016	21:03:00.950	7KP	Bid market	106.470	1	–
⋮	⋮	⋮	⋮	⋮	⋮	⋮
10 June 2016	20:59:20.148	HT6	Buy Limit	107.405	3	20:59:29.072

**Table 2:** Abbreviations for eight types of order events. Note the six types of orders in the order book do not include the orders of target HFTs. Therefore, $\left\{t_{i}\right\}$ differs for each HFT.

**TS**	:	Sell limit of the target HFT itself	**TB**	:	Buy limit of the target HFT itself
**SL**	:	Sell limit in order book	**BL**	:	Buy limit in order book
**SC**	:	Sell cancel in order book	**BC**	:	Buy cancel in order book
**HS**	:	Hit sell	**HB**	:	Hit buy

**Table 3:** Mean values of the estimated time constant ${\hat{\beta }}_{n,m}^{-1}$ (s) for the HFTs in Group A.

	m	TS	TB	SL	BL	SC	BC	HS	HB
n									
TS		0.102	0.295	0.076	0.087	0.081	0.069	0.110	0.404
TB		0.118	0.099	0.114	0.071	0.066	0.091	0.148	0.111

**Table 4:** Sample means of 16 time constants, $\beta_{n,m}^{-1}$(S), for HFTs in Group B.

	m	TS	TB	SL	BL	SC	BC	HS	HB
n									
TS		0.099	0.100	0.681	0.109	0.110	0.285	0.101	0.099
TB		0.099	0.100	0.259	0.373	0.558	0.105	0.099	0.101

**Table 5:** Sample means of 16 time constants, $\beta_{n,m}^{-1}$(S), for HFTs in Group C.

	m	TS	TB	SL	BL	SC	BC	HS	HB
n									
TS		0.134	17.781	0.266	0.099	0.096	0.359	0.109	0.154
TB		11.201	0.098	0.269	0.258	0.355	0.094	0.132	0.103

## Data Availability

Restrictions apply to the availability of these data. Data were obtained from the EBS. We obtained permission for publication.
